# *Itm2a* Is a Pax3 Target Gene, Expressed at Sites of Skeletal Muscle Formation *In Vivo*

**DOI:** 10.1371/journal.pone.0063143

**Published:** 2013-05-01

**Authors:** Mounia Lagha, Alicia Mayeuf-Louchart, Ted Chang, Didier Montarras, Didier Rocancourt, Antoine Zalc, Jay Kormish, Kenneth S. Zaret, Margaret E. Buckingham, Frederic Relaix

**Affiliations:** 1 Département de Biologie du Développement, CNRS URA 2578, Institut Pasteur, Paris, France; 2 UPMC Paris 06, UMR-S 787, Paris, France; 3 INSERM, Avenir team, Pitié-Salpétrière, Paris, France; 4 Institut de Myologie, Paris, France; 5 Department of Cell and Developmental Biology, University of Pennsylvania School of Medicine, Philadelphia, Pennsylvania, United States of America; University of Minnesota Medical School, United States of America

## Abstract

The paired-box homeodomain transcription factor Pax3 is a key regulator of the nervous system, neural crest and skeletal muscle development. Despite the important role of this transcription factor, very few direct target genes have been characterized. We show that *Itm2a,* which encodes a type 2 transmembrane protein, is a direct Pax3 target *in vivo*, by combining genetic approaches and *in vivo* chromatin immunoprecipitation assays. We have generated a conditional mutant allele for *Itm2a*, which is an imprinted gene, by flanking exons 2–4 with loxP sites and inserting an *IRESnLacZ* reporter in the 3′ UTR of the gene. The *LacZ* reporter reproduces the expression profile of *Itm2a*, and allowed us to further characterize its expression at sites of myogenesis, in the dermomyotome and myotome of somites, and in limb buds, in the mouse embryo. We further show that *Itm2a* is not only expressed in adult muscle fibres but also in the satellite cells responsible for regeneration. *Itm2a* mutant mice are viable and fertile with no overt phenotype during skeletal muscle formation or regeneration. Potential compensatory mechanisms are discussed.

## Introduction

*Pax* genes, which encode paired domain transcription factors, play key roles in tissue specification and organogenesis during embryonic development [1]. *Pax3* mutant embryos have defects in neural tube closure, severely reduced neural crest migration in the trunk and skeletal muscle defects. Mesodermal expression of *Pax3* is first detected in presomitic mesoderm and then in somites where it becomes restricted to the dorsal dermomyotome. Multipotent Pax3-positive cells of the dermomyotome give rise to a number of derivatives, including the skeletal muscle of the trunk and limbs. Cells, that have activated the myogenic determination genes, *Myf5* and *Mrf4*, delaminate from the edges of the dermomyotome to form the first muscle mass, the myotome, beneath the dermomyotome. At limb level, Pax3-positive cells, that have not yet entered the myogenic program, migrate into the limb bud where they provide the progenitor cell pool for skeletal myogenesis. In the absence of Pax3, limb muscles are absent, cells fail to migrate from the hypaxial dermomyotome and this domain of the somite undergoes apoptosis. Pax7, a closely related paralogue of Pax3, is also expressed in the central domain of the dermomyotome, as well as in myogenic progenitors when they reach the limb. As the somite matures, the central domain of the dermomyotome loses its epithelial structure and Pax3/Pax7-positive myogenic progenitors enter the underlying muscle mass of the myotome, which later expands and segments to give rise to the muscles of the trunk. The Pax3/Pax7 population provides a reserve of myogenic progenitors for all subsequent muscle growth. In the *Pax3/Pax7* double mutant, these cells fail to enter the myogenic program, and many of them die [2]. This population is also the source of postnatal myogenic progenitors, known as satellite cells because of their characteristic position under the basal lamina of the muscle fiber [3], [4]. Pax7 marks the majority of satellite cells [5], many of which also continue to transcribe *Pax3* [6]. In addition to contributing to post-natal growth, these cells are also the main source of progenitors for adult muscle regeneration [7], [8]. In the adult, satellite cells are mainly quiescent, undergoing activation on injury when they express the myogenic determination factors Myf5 and MyoD, proliferate and then differentiate to form new fibers. As in the embryo, the differentiation process is initiated by expression of the myogenic differentiation factor, Myogenin, and cell cycle withdrawal. *Pax7/3* are down-regulated prior to the onset of differentiation, or remain expressed in cells that reconstitute the satellite cell pool.

Pax3, and later also Pax7, thus plays a key role in skeletal myogenesis [1]. Mutant phenotypes indicate its function in this context in the embryo, - in somitogenesis, delamination and migration of cells from the dermomyotome, cell survival/proliferation and the entry of progenitor cells into the myogenic program -, yet very few direct Pax3 target genes have been identified. The gene encoding the tyrosine kinase receptor, *c-Met*, has been described as a Pax3 target [9] and *c-met* mutants lack all muscles of migratory origin [10]. A critical enhancer element, upstream of the *Myf5* gene, is directly activated by Pax3 [11] and Pax7 targets a regulatory sequence of the *MyoD* gene [12]. Since the binding sites for Pax3 and Pax7 are similar [13], both factors probably activate common targets, as shown for Pax7 on the *Myf5* enhancer [14]. *Fgfr4*, involved in the self-renewal versus differentiation of muscle progenitors, is also directly regulated by Pax3 through a 3′ myogenic enhancer element [15]. Other potential targets have come from genetic screens. Since cells tend to undergo apoptosis in the absence of Pax3, gain of function rather than loss of function comparisons have been particularly valuable. PAX3 and PAX7 are implicated in Alveolar Rhabdomyosarcoma (ARMS), a pediatric tumour of skeletal muscle origin that results from a translocation between PAX3/7 and FKHR (FOXO1A). This generates a hybrid transcription factor, PAX-FKHR, which binds and transactivates PAX3/7 target genes, leading to their over-expression [16]. A number of screens based on over-expression of Pax3/7 in cultured cells have been carried out [17], [18]. CASTing experiments with cyclic amplification and selection of genomic sequences bound by PAX3, PAX3-FKHR [19] or a large scale ChIP-seq screen for PAX3-FKHR binding sites in ARMS cells [20] have also led to lists of potential targets. We constructed a *Pax3^PAX3-FKHR/+^* allele and showed that in *Pax3^PAX3-FKHR/+^* embryos, *c-met* for example, as well as *Myf5* and *MyoD*, are up-regulated [21]. This allele rescues the *Pax3* mutant phenotype and we therefore devised a screen incorporating a *Pax3^GFP^* allele [21] which permits us to purify the Pax3-positive population by flow cytometry. Comparison of the transcriptome of somites at E9.5 and forelimb buds at E10.5 of *Pax3^GFP/+^* and *Pax3^GFP/PAX3-FKHR^* embryos led to the identification of genes that are up- or down-regulated in the presence of FKHR [22]. These included known Pax3 targets and genes such as *Foxc2*, implicated in cell fate decisions in the dermomyotome [23], which is negatively regulated by *Pax3*. Among the genes that are up-regulated (2.79, 3.27 fold in limb buds and somites, respectively) on the gain of function, *Pax3^PAX3-FKHR^*, background in our *in vivo* screen was *Itm2a*, also identified in several of the *in vitro* screens. In this study, we show that *Itm2a* is a direct Pax3 target *in vivo* and examine its role in myogenesis.

*Itm2a* is expressed in the C2C12 muscle cell line where its mRNA increases on differentiation and over-expression leads to muscle creatine kinase up-regulation and more myotube formation [24]. This is in contrast to the chondrocyte model where over-expression of Itm2a delayed the onset of differentiation [25]. Expression studies in cell lines suggest that Itm2a is associated with early stages of the chondrogenic program.

*Itm2a* encodes a transmembrane protein, which, in addition to a transmembrane domain, has a Brichos domain of about 100 amino acids, potentially acting as a chaperone domain [26]. This Brichos domain has been identified in several previously unrelated proteins that are linked to major human diseases such as dementia, respiratory distress and cancer [27]. The Brichos domain is potentially associated with anti-apoptotic functions. Mutation of the Brichos domain of a surfactant protein (SP-C) causes proteasome dysfunction and caspase 3/4 activation leading to apoptosis [28].

In this study, we have investigated the expression and potential role of Itm2a during myogenesis in the mouse embryo and during muscle regeneration in the adult. We establish that *Itm2a* lies genetically downstream of *Pax3* and confirm that it is a direct target by ChIP assays on embryonic extracts. In order to investigate function we made a conditional mutant allele of *Itm2a*, incorporating an *nLacZ* reporter. The expression profile of the reporter confirms that *Itm2a* is transcribed at sites of myogenesis, both in the embryo and the adult. The *Itm2a* mutant has no detectable myogenic phenotype.

## Results

### *Itm2a* Expression is Modulated by Pax3 and Myf5 during Development

**Figure 1:**
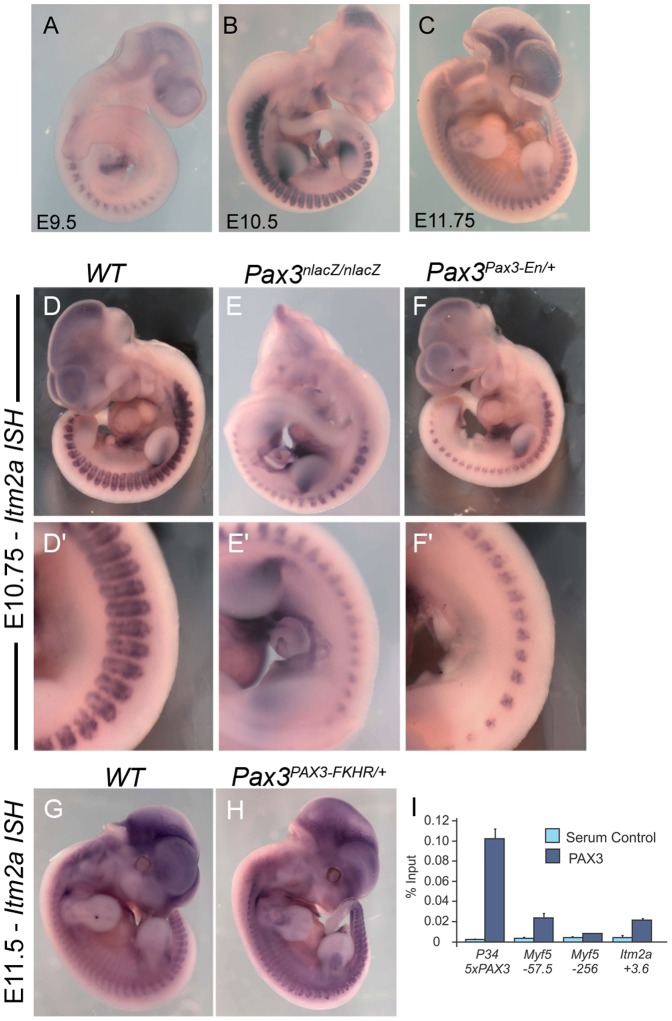
*Itm2a* is a novel Pax3 target. A–C, Whole mount *in situ* hybridization with an *Itm2a* antisense riboprobe at E9.5 (A), E10.5 (B) and E11.75 (C). D–F’, Whole mount *in situ* hybridization with an *Itm2a* antisense riboprobe on wild type (WT) (D, D’), *Pax3^nLacZ/nLacZ^* (E, E’) and *Pax3^Pax3-En/+^* (F, F’) embryos at E10.5. D’–F’ show close-ups of the interlimb somite region. As expected, the modification of *Itma* transcription in the mutant is restricted to Pax3-expressing cells. G–H, Whole mount *in situ* hybridization with an *Itm2a* antisense riboprobe on wild type (WT) and *Pax3^PAX3-FKHR/+^* embryos at E11.5. I, Real-time quantitative PCR using primers for the putative Pax3 binding site identified in [19] in the Itm2a sequence at +3.6 kb. The 5 Pax3-binding sites in the P34 transgene [21] and a functional Pax3 site at −57.5 kb from the Myf5 gene [11] provide positive controls. A Myf5 flanking sequence at −256 kb that does not bind Pax3 [11] provides a negative control. Results are expressed as a percentage of PCR signal on input DNA, showing enrichment after Pax3 immunoprecipitation, or with an IgG antibody.

The gene encoding the transmembrane protein Itm2a emerged as a potential Pax3 target in myogenic progenitors in the mouse embryo [22]. To characterize *Itm2a* expression during mouse development, whole-mount *in situ* hybridization was performed on wild-type mouse embryos from Embryonic day (E) 9.5 to 11.75 (Figure 1 A–C). At E9.5, expression of *Itm2a* is detected in the somites, heart and brain (Figure 1A). Expression at these sites is maintained at E10.5 and E11.75, with additional expression in the forelimb bud at sites of chondrogenesis at E10.5 and of myogenesis, as clearly distinguished at E11.75 (Figure 1B–C), as well as in the pharyngeal arches (Figure 1B). The sites of somitic expression (Figure 1 and data not shown) suggest expression in the dermomyotome as well as in the myotome.

In order to validate genetically that *Itm2a* is a Pax3-regulated gene, we perfomed *in situ* hybridization on embryos carrying modified *Pax3* alleles.

In *Pax3*-mutant embryos, *Itm2a* expression is strongly reduced (Figure 1E–E’), however, apoptosis in the epaxial and, notably, the hypaxial dermomyotome of the somite complicates interpretation [1]. In the presence of the allele for *Pax3^Pax3-En^* which encodes a fusion protein in which the DNA binding domain of Pax3 is fused to the Engrailed repressor domain, heterozygote *Pax3^Pax3-En/+^* embryos have a partial loss of Pax3 function [11]. In these embryos *Itm2a* expression is severely impaired at E10.5 in the somitic region (Figure 1F–F’), a phenotype readily seen from E9.5 (data not shown). At these early stages, most Pax3-positive cells are still present in the somite of *Pax3^Pax3-En/+^* embryos, excluding this reduced expression as secondary to loss of cells [11] (Figure S1). Moreover, this epistatic regulation is specific since the expression of *Itm2a* is only affected at sites of *Pax3* expression in our mouse models (Fig. 1E–F’). Conversely, in the presence of a *Pax3^PAX3-FKHR^* gain of function allele, which encodes a fusion protein in which the DNA binding domain of the human PAX3 protein is fused to the transcriptional activation domain of FKHR (FOXO1A) [21], *Itm2a* expression is increased in the somites, and in the skeletal muscle cells of the limbs of *Pax3^PAX3-FKHR/+^* embryos as shown at E11.5 (Figure 1G–H, Figure S2). Altogether, our data validate the results of the transcriptome analysis [22] and confirm that *Itm2a* limb-expression is not restricted to sites of chondrogenesis but that it is also expressed at sites of myogenesis in the proximal limb bud (ex. Figure 1H).

Residual expression of *Itm2a* in the absence of Pax3 (Figure 1E) suggests that Pax7 may also activate this gene. To test this hypothesis, *Itm2a* expression was evaluated in embryos where *Pax7-IRESnLacZ* replaces *Pax3*. In *Pax3^Pax7-IRESnLacZ/Pax7-IRESnLacZ^* embryos, Pax7 is able to substitute for all Pax3 functions during trunk myogenesis but specific defects are observed in the limbs [13]. In the absence of endogenous Pax3, and in the presence of additional Pax7 from the *Pax3* allele in *Pax3^Pax7-IRESnLacZ/Sp^* mice, somitic expression of *Itm2a* is decreased, notably at the hypaxial level where Pax3 plays a critical role [1] (Figure S3). However, since some expression is still detected in the absence of Pax3, we conclude that *Itm2a* lies genetically downstream of both *Pax3* and *Pax7* at sites of myogenesis, but that Pax3 is a stronger activator of its expression.

Since *Itm2a* is expressed in developing skeletal muscle, we analyzed its expression in *Myf5^nLacZ/nLacZ^* mice in which the myogenic determination factors Myf5 and Mrf4 are absent [29], [30]. In these mutant embryos, myogenic progenitor cells are blocked at the edges of the dermomyotome and no myotome is formed until the activation of *MyoD*, from E11.5 [31]. In the absence of myogenesis, expression of *Itm2a* is barely detectable at E10.75 (Figure S4) and strongly reduced at E11 (Figure S4). These data therefore suggest that *Itm2a* is regulated by both Pax3 and Myf5/Mrf4, which have parallel functions in the establishement of the primary myotome [31], or that *Itm2a* expression in the somite by this stage is mainly in the differentiating muscle cells of the myotome, which start to be rescued by *MyoD* expression in the mutant from E11.

### *Itm2a* is a Direct Pax3 Target Gene *in vivo*

Genetic studies therefore suggest that *Itm2a* is a downstream target of Pax3/7 but do not demonstrate whether this regulation is direct. A putative Pax3 binding site has been reported in the first intron of the *Itm2a* gene and gel mobility shift assays suggested that Pax3 can directly bind this sequence *in vitro* and transactivate it in cell lines [19]. We tested this putative binding site for Pax3 binding *in vivo* using chromatin immunoprecipitation (ChIP) on chromatin from crosslinked cells within pools of E11.5 embryonic trunk and limbs. (Figure 1I) [15]. Results of quantitative PCR using this binding site, compared to the 5 Pax3 binding sites of the *P34* transgene [21] or the binding site of the *Myf5* enhancer at −57.5 Kb, as positive controls [11], shows that Pax3 enrichment of the *Itm2a* sequence is approximately equivalent to that of the *Myf5* −57.5 kb sequence. This demonstrates that this region in the first intron of *Itm2a* is bound by the Pax3 protein *in vivo*.

The antibody used for immuno-precipitation can also recognize Pax7, therefore, as suggested by the genetic studies (Figure S3), Pax3 binding sites located in the first intron of *Itm2a* may also be bound by Pax7. Recent Chip-Seq experiments, performed on cultured myoblasts also reveal Pax7 binding to *Itm2a* (however these are not located within the 1st intron) [32].

### An *Itm2a* Conditional Allele Incorporating a LacZ Reporter

**Figure 2:**
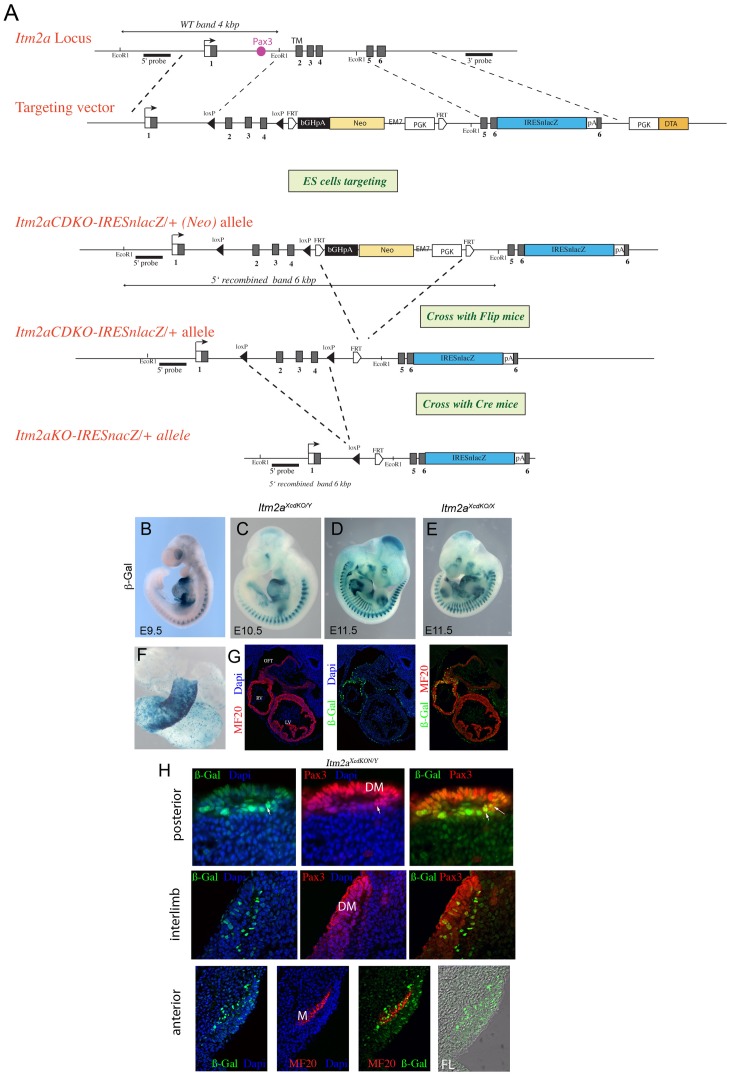
Generation of an *Itm2a* conditional reporter allele. A. Schematic diagram of the *Itm2a* locus and targeting construct. The construct contains *loxP* sites inserted into exon 1 and 4. A *PGK-Neo-pA* (*Neo*) selection marker is flanked by FRT sites and inserted into exon 4. An *IRES-nLacZ* cassette has been inserted into the 3′ UTR (exon 6). A counter-selection cassette encoding the A subunit of Diphtheria Toxin was inserted at the 5′ end of the targeting vector. Schematic diagrams of the *Itm2a^cdKO-IRESnLacZ (Neo)^* and *Itm2a^KO-IRESnLacZ (Neo)^* alleles are also shown. Probes and restriction enzymes are indicated, with the size of the resulting wild-type and recombined restriction fragments. Pax3 sites located in *Itm2a* intron1 are represented by a pink box. B–D, X-Gal stained *Itm2a^XcdKO/Y^* embryos at E9.5 (B), E10.5 (C) and E11.5 (D). E, An X-Gal stained *Itm2a^XcdKO/X^* embryo at E11.5. Note the chimeric expression of the reporter, due to random *X* inactivation. F, An isolated X-Gal stained heart of an *Itm2a^XcdKO/Y^* embryo at E9.5. G, Immunohistochemistry on transverse sections through the heart region of an E9.5 *Itm2a^XcdKO/Y^* embryo using antibodies recognizing striated muscle myosin MF20 (red), β-Gal (green). Dapi staining of nuclei is in blue. The right hand panel shows a merged image with both antibodies. OFT, outflow tract; RV, right ventricle; LV, left ventricle. H, Immunohistochemistry on transverse sections through immature somites of an *Itm2a^XcdKO/Y^* embryo at E10.5, in the more caudal region (top), in the interlimb region (middle) and just anterior to the forelimb (bottom), using antibodies recognizing Pax3 or striated muscle myosin (MF20) (red) as indicated on the Figure, and β-Gal (green). Dapi staining of nuclei is in blue. Merged images are shown on the right. In the bottom series, the right hand panel shows phase contrast with β-Gal on a somite just anterior to the forelimb bud. Dermomyotome (DM), Myotome (M), Forelimb (FL). Arrows point to cells expressing Pax3 and ßGal (Itm2a).

In order to study Itm2a function *in vivo*, the *Itm2a* gene was targeted by homologous recombination. A conditional approach has been adopted to avoid potential precocious embryonic lethality. *LoxP* sites were introduced before and after exons 2–4 so that the transmembrane domain is removed and the Brichos domain is compromised on crossing with a mouse line expressing Cre recombinase. An *IRESnLacZ* reporter, which encodes β-Galactosidase (β-Gal), placed downstream of an internal ribosome entry site (IRES), has also been inserted into the 3′UTR of the gene, in order to follow *Itm2a* expressing or mutant cells. The targeting strategy is illustrated in the scheme shown in Figure 2A. The Pax3 binding site is located 5′ to the floxed region.

After generation of chimaeric mice and germline transmission, adult males or females carrying either the conditional *Itm2a^XcdKON^* (which contains the *Neo* selection cassette) or *Itm2a^XcdKO^* (with the *Neo* selection cassette removed) alleles, were viable and fertile and did not present any obvious phenotype. *Itm2a* is located on the X chromosome, which is why a nomenclature showing the X/Y genotype has been adopted in this study: *Itm2a^XcdKO/Y^* for male and *Itm2a^XcdKO/XcdKO^* for female embryos.

In order to validate the expression profile from the *IRESnLacZ* reporter, the X-Gal profile of *Itm2A^XcdKO/Y^* male embryos from E9.5 to E11.5 was compared to endogenous *Itm2a* expression. As shown at E9.5, E10.5 and E11.5, β-Galactosidase activity from the *nLacZ* reporter (Figure 2B–D) follows endogenous *Itm2a* expression (Figure 1). Strikingly, *Itm2A^XcdKO/X^* female embryos show a chimaeric expression of the *nLacZ* reporter at all sites of *Itm2a* expression, as shown at E11.5 (Figure 2E, see also Figure S5). This phenomenon is probably due to random X-inactivation in female embryos (reviewed in [33]), resulting in a mosaic expression of the wild-type and engineered alleles (Figure S5). In the case of a conditional mutated allele, the recombined allele should not significantly perturb the inactivation pattern, and indeed previous gene targeting in mice already reported mosaicism of reporter expression in female embryos (for example with *Sox3* targeting [34]). Altogether these results validate the targeting of the *Itm2a* locus, showing that the *nlacZ* reporter follows endogenous *Itm2a* gene expression, with random X-inactivation in female embryos.

In addition to skeletal muscle, the expression of *Itm2a* was documented previously in two main tissues: bone [35] and thymus [36]. In the developing skeletal system, *Itm2a* expression has been reported in areas undergoing endochondral ossification, more specifically in chondrocytes of the resting and proliferating zones [37], in keeping with the profile of *nLacZ* expression that we observe (Figure S6). The *nLacZ* reporter also reveals other sites of expression, such as the developing heart, where β-Galactosidase positive cells are notably present in the outflow tract and right ventricle, which are derivatives of the anterior part of the second heart field [38] (Figure 2F–G).

In order to determine in which cells of the developping somite *Itm2a* is expressed, and because we could not find a reliable antibody against Itm2a protein that works for immunohistochemistry, we relied on the β-Galactosidase (β-Gal) protein to follow Itm2a expression. At an early stage of somite development, in more posterior somites at E10.5, β-Gal (Itm2a) expression is detected in the dermomyotome marked by Pax3, and in the underlying cells of the myotome that are undergoing skeletal muscle differentiation (Figure 2H upper panels). In more developed interlimb somites and in anterior somites, β-Gal (Itm2a) expression is restricted to a subset of cells in the dermomyotome as well as the myotome (Figure 2H middle and lower panels).

### *Itm2a* is Dispensable for Embryonic Development and Myogenesis

**Figure 3:**
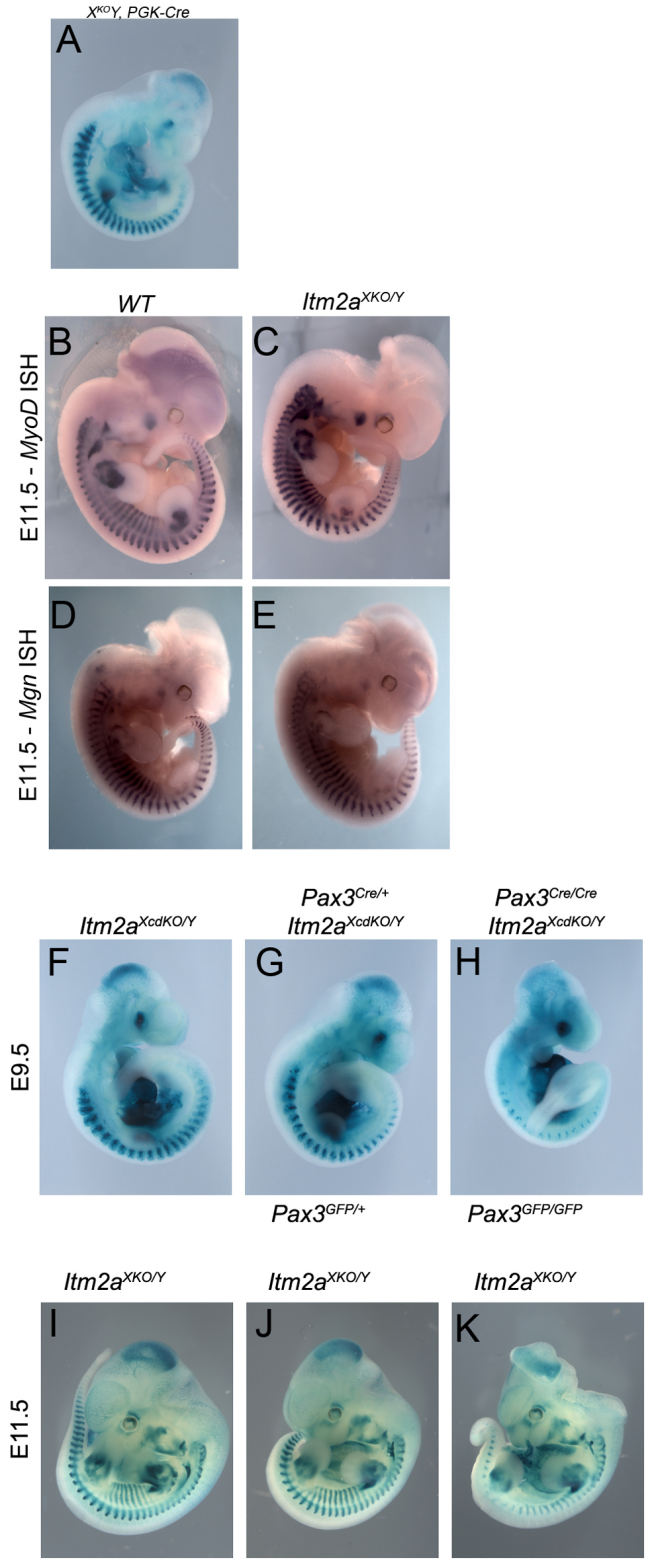
Phenotypic analysis of *Itm2a* mutant embryos. A, X-Gal staining of an *Itm2a^XKO/Y^; PGK-Cre* embryo at E10.5. B–E, Whole mount in situ hybridization with a MyoD (B–C) and Myogenin (D–E) antisense riboprobe on control (WT) (B, D) and Itm2aXKO/Y embryos at E11.5. In A, C and E the mice were crossed with the PGK-Cre line to delete the floxed Itma2 allele. F–H, X-Gal staining of *Itm2a^XcdKO/Y^* (F), *Itm2a^XcdKO/Y^*; *Pax3^Cre/+^* (G) and *Itm2a^XcdKO/Y^*; *Pax3^Cre/Cre^* (H) embryos at E9.5. I–J, X-Gal staining of *Itm2a^XKO/Y^* (I), *Itm2a^XKO/Y^*; *Pax3^GFP/+^* (J) and *Itm2a^XKO/Y^*; *Pax3^GFP/GFP^* (K) embryos at E11.5.

As shown above, we designed the *Itm2a^XcdKO^* allele so that it can be used to generate an *Itm2a* loss of function allele when intercrossed with mice expressing ubiquitous or tissue-specific *Cre* drivers. When *Itm2A^XcdKO/Y^* adult males were crossed with females carrying a maternally-expressed *PGK-Cre* transgene [39], we obtained viable *Itm2A^XKO/Y^* adult males and *Itm2A^XKO/X^* adult females in mendelian ratios. *Itm2A^XKO/Y^*; *PGK-Cre* male embryos expressed the *nLacZ* reporter at all sites of *Itm2a* expression, as shown at E10.5 (Figure 3A, Figure S7) and had no obvious phenotype. We also intercrossed *Itm2A^XKO/Y^* adult males and *Itm2A^XKO/X^* adult females to generate *Itm2A^XKO/XKO^* adult females, and again these animals were viable and fertile, with no evident phenotype. Next, we analyzed the expression of the myogenic determination gene, *MyoD*, and the myogenic differentiation gene, *Myogenin*, by *in situ* hybridization in *Itm2A^XKO/Y^* male (Figure 3B–E) and *Itm2A^XKO/XKO^* female embryos (data not shown). In both cases, myogenesis was not affected by the loss of *Itm2a* expression. In order to evaluate whether this absence of phenotype was due to an early compensatory mechanism, we mutated *Itm2a* at later stages, specifically in the myogenic lineage by crossing *Itm2A^XcdKO/Y^* adult males with *Pax3^Cre/+^* females carrying an allele of *Pax3* targeted with the *Cre recombinase* sequence [40]. *Itm2A^XcdKO/Y^*; *Pax3^Cre/+^* males have a deletion of *Itm2a* in all Pax3-derived cell lineages, including all skeletal muscle of the trunk and limbs, the dorsal neural tube, and all neural-crest derived tissues [40]. *Itm2A^XcdKO/Y^*; *Pax3^Cre/+^* males were viable, fertile and did not show any detectable phenotypes during development (Figure 3F–G). Finally, as *Itm2a* is a direct Pax3 target gene, we tested whether *Pax3* and *Itm2a* interact genetically. *Itm2A^XcdKO/Y^*; *Pax3^Cre/+^* males were crossed with *Pax3^Cre/+^* females to generate *Itm2A^XcdKO/Y^*; *Pax3^Cre/Cre^* male double mutant embryos in the Pax3 lineage (Figure 3H), and *Itm2A^XKO/Y^*; *Pax3^GFP/+^* males were crossed with *Pax3^GFP/+^* females to generate *Itm2A^XKO/Y^*; *Pax3^GFP/GFP^* male double mutant embryos (Figure 3J). In both cases, the double-deficient embryos displayed a classic *Pax3* mutant phenotype, but no additional phenotypes were observed (Figure 3 F–K, Figure S8).

### *Itm2a* is Expressed in Adult Skeletal Muscle

In adult mice, assay of *Itm2a* transcripts by Northern blot had shown that these are highest in the thymus and also expressed, at lower levels, in lymph nodes, spleen, brain, heart, lung, stomach and uterus [36]. *Itm2a* transcripts also accumulate in adult skeletal muscle, where the protein has been detected previously [41]. The link with Pax3/7 suggests that *Itm2a* might be expressed in adult skeletal muscle progenitor cells (satellite cells), however *Itm2a* expression had only been reported in muscle fibers [24]. The β-Gal reporter in our *Itm2a* allele provides a robust system to identify the cell types that transcribe *Itm2a* in adult skeletal muscle. First, we perfomed X-Gal staining on sections of EDL and Diaphragm skeletal muscle from adult *Itm2A^XcdKO/x^* mice. The reporter was expressed in nuclei located under the basal lamina of the muscle fiber, as well as in myonuclei within most fibers (90% in EDL, 64% in Diaphragm for both locations). We also detected expression in glial cells surrounding nerves (data not shown).

**Figure 4:**
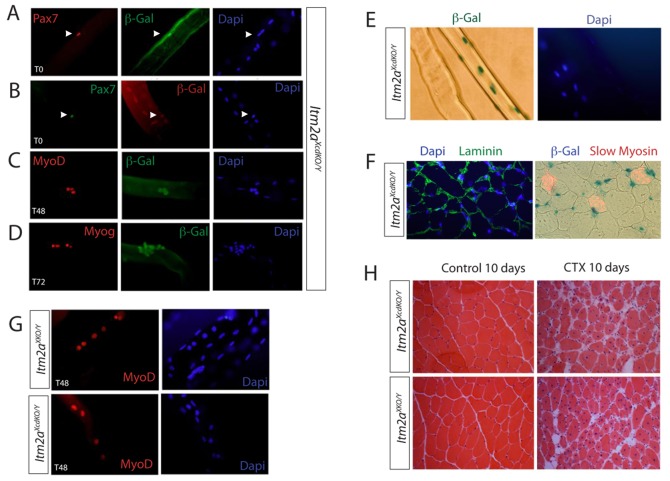
*Itm2a* is expressed in adult skeletal muscles. A–D, Immunohistochemistry on isolated single fibers from the EDL muscle of adult *Itm2a^XcdKO/Y^* mice immediately after isolation (T0) (A, B), or after 48 h, (T48) (C) or 72 h (T72) (D) culture, using Dapi staining (blue) and antibodies recognizing Pax7 (red, A, green B), β-Gal (green, A, C, D, red, B), MyoD (red, C) and Myogenin (red, D). Arrowheads point to Pax7-positive satellite cells (A,B). E, X-Gal staining for β-Gal on isolated single EDL fibers from adult *Itm2a^XcdKO/Y^* mice at T0 showing examples where the myonuclei are positive or negative. Dapi is shown in blue. X-Gal positive staining quenches DAPI. F, Immunohistochemistry on transverse sections of EDL muscle isolated from adult *Itm2a^XcdKO/Y^* mice with Dapi staining (blue), and antibodies recognizing Laminin (green) (left) or slow myosin heavy chain (red) and β-Gal (blue) (right) as indicated. G, Immunohistochemistry experiments on isolated single fibers from adult *Itm2a^XcdKO/Y^* and *Itm2a^XKO/Y^* mice after 48 h culture (T48) with Dapi (blue) and an antibody recognizing MyoD (red). H, Eosin-hematoxylin staining on transverse sections of the EDL muscle isolated from adult control Itm2aXcdKO/Y (top) and Itm2aXKO/Y (bottom) mice, 10 days after cardiotoxin injury (CTX) (right) or PBS injection (control) (left). Dapi staining (blue) marks nuclei. In G and H the mice were crossed with the PGK-Cre line to delete the floxed Itma2 allele.

To examine more precisely the expression of *Itm2a* during adult myogenesis, we used a single fiber culture system [42]. We isolated single fibers from the EDL muscle of adult (8 weeks) *Itm2A^XcdKO/Y^* mice and fixed them immediately (T0). After immunohistochemistry, we detected the expression of the β-Gal reporter in quiescent satellite cells marked by Pax7 expression, at T0 (Figure 4A). Thus, Itm2a is a novel satellite cell marker. In addition to satellite cells, most fibers also showed expression of the β-Gal reporter in myonuclei (Figure 4B and E). This was also observed in other muscles examined (data not shown and see below). When isolated single fibers were maintained in culture for 48 h (T48) and 72 h (T72) (Figure 4D), the β-Gal reporter colocalized with MyoD (Figure 4C) and Myogenin (Figure 4D), demonstrating that *Itm2a* expression is maintained in activated, proliferating (MyoD-positive) and differentiating (Myogenin-positive), satellite cells.

Since *Itm2a* expression is detected within a subset of muscle fibers (Figure 4E), we investigated whether this expression was linked to the fiber type. The diaphragm contains mostly slow- and intermediate-twitch muscle fibers (types I and II, respectively). We therefore used a slow myosin antibody that detects type I fibers to perform colocalization experiments on diaphragm sections from *Itm2a^XcdKO/Y^* adult mice. A laminin antibody was used to outline the fibers and Dapi to mark nuclei. As shown in Figure 4F, we could not detect a correlation between the expression of the β-Gal reporter and the fiber type.

Finally, we investigated whether *Itm2a* could play a role during adult myogenesis. We isolated single fibers from *Itm2a^XcdKO/Y^* (control) and *Itm2a^XKO/Y^* (mutant) adult mice and performed single fiber culture experiments. Mutant as well as control satellite cells expressed MyoD within 24 h after activation as previously described [42] (results not shown), and after 48 h of culture (T48) (Figure 4G). No difference in behaviour could be detected in satellite cells with or without Itm2a. We also investigated whether Itm2a is involved in skeletal muscle regeneration. We performed Cardiotoxin-mediated injury of the EDL muscle and analyzed the regeneration process 10 days later. *Itm2a^XcdKO/Y^* (control) and *Itm2a^XKO/Y^* (mutant) adult mice displayed a similar extent of skeletal muscle regeneration, as evidenced by the formation of new fibers, maked by centrally located nuclei (Figure 4H).

We conclude that Itm2a marks satellite cells and myonuclei of most fibers but is dispensable for adult myogenesis.

## Discussion

In this investigation of the role of Itm2a during skeletal muscle formation *in vivo*, we demonstrate that the Pax3 binding site in the first intron of the *Itm2a* gene [19] binds Pax3 *in vivo*, as demonstrated by ChIP experiments with embryonic extracts. We therefore conclude that *Itm2a* is a direct Pax3 target. The introduction of an *nLacZ* reporter into an allele of *Itm2a* permitted us to confirm the myogenic sites of expression, suggested by *in situ* hybridization with an *Itm2a* probe in the embryo. *Itm2a* is initially expressed in the Pax3-positive multipotent cells of the dermomyotome. Subsequently, it is mainly expressed in the differentiating muscle cells of the underlying myotome. In these cells, that no longer express Pax3, the myogenic determination factors, Myf5 and Mrf4, may maintain its transcription, suggested by the loss of *Itm2a* expression in the *Myf5/Mrf4* mutant, as in the case of *Fgfr4* [15]. Indeed E-box sequences (CANNTG) that can bind myogenic factors are also present in the sequence in the first intron of *Itm2a*, that binds Pax3 (results not shown). *Itm2a* is also expressed in the developing muscle masses of the limb bud and at other sites of myogenesis. *Itm2a* expression persists in the satellite cells of adult skeletal muscle, both in quiescent and activated states. Myonuclei in most muscle fibers remain β-Gal positive - a phenomenon which is not related to fiber type. This kind of heterogeneity, at present unexplained, was also seen in the sites of expression directed by the *A17 Myf5/Mrf4* enhancer in adult muscle [43]. In mutant embryos in which Pax7 replaces Pax3 and in muscle satellite cells of the EDL muscle which are Pax7-positive, but not Pax3-positive, *Itm2a* is transcribed, indicating that both Pax transcription factors can activate the gene and indeed *Itm2a* continues to be expressed in both *Pax3* and *Pax7* (results not shown) mutant embryos. When these Pax factors are highest, in quiescent satellite cells, *Itm2a* transcripts are maximal [44]. In addition to sites of myogenesis and other known sites of *Itm2a* expression, we document expression in the developing heart in the outflow tract and right ventricle, which derive from the anterior second heart field [38].

In the conditional *Itm2a* mutant embryos that we have constructed, with both early (*PGK-Cre*) and Pax3-specific (*Pax3^Cre/+^*) activation of the Cre recombinase, there is no detectable phenotype in the mutants. All aspects of embryonic myogenesis take place as in wild type embryos and this is also the case for adult satellite cell behaviour in culture or during skeletal muscle regeneration. The conditional mutation eliminates most of the coding exons of the *Itm2a* gene, including the transmembrane and a large part of the Brichos domain. RT-PCR analysis indicates that a truncated transcript is produced, as indeed shown by continuing expression of the *IRES-nLacZ* reporter integrated into the 3′ UTR of the gene. It is very unlikely that this truncated transcript leads to the accumulation of a partial Itm2a protein that can compensate for the function of Itm2a. An alternative explanation of the lack of a phenotype lies in potential compensation by other members of the *Itm2a* gene family. Itm2b and Itm2c share 38–45% homology with Itm2a, mainly in the -COOH terminal domain. Expression of *Itm2c* is restricted to the brain [45], whereas *Itm2b* is ubiquitously expressed [46]. In a differential screen on Interleukin 2 (IL-2) deprived or stimulated cells, *Itm2b* emerged as a gene induced on IL-2 deprivation [47]. Further analysis showed that Itm2b has a BH-3 domain that characterizes Bcl2 family members, in which this domain is essential for induction of apoptosis. Itm2b interacts with Bcl2 *in vitro*. It is unclear whether this pro-apoptotic function is shared by Itm2a, however when the coding sequences are compared no conservation of the BH-3 domain was observed. In contrast the Brichos domain, potentially associated with anti-apoptotic functions [28] is highly conserved. In *Itm2a* mutants, we did not detect transcripts of *Itm2c* or any up-regulation of *Itm2b*, the expression of which is at a barely detectable level, at sites of myogenesis in the embryo, although both *Itm2c* and *Itm2b* transcripts are detected with transcripts for *Itm2a* in quiescent satellite cells [44]. It is of course possible in the embryo that the basal level of *Itm2b* transcription, or the presence of other proteins with a Brichos domain or with other features of Itm2a, compensate for the absence of Itm2a. Itm2a and related transmembrane proteins may well play a role as adaptors in promoting membrane receptor function and/or in transmitting signals either through intracellular pathways that connect with the Brichos domain, or directly by the intervention of Itm2a intracellular domains after cleavage on the membrane. Further investigation of the proteins that interact with Itm2a at sites of myogenesis *in vivo* will be required to throw more light on the role of Itm2-like proteins during myogenesis.

In conclusion, we have shown that *Itm2a* is a direct Pax3 target *in vivo* during myogenesis in the mouse embryo. Moreover, during adult skeletal myogenesis, Itm2a is a new satellite cell marker. Finally, we have generated a conditional floxed allele of *Itm2a* which is available for further genetic re-combination studies.

## Materials and Methods

### Ethical Statement

All experiments with mice reported in this paper were performed in the Monod animal house of the Pasteur Institute, under the control of individuals with an animal experimentation license, according to the regulations of the French Ministry of Agriculture. The Monod animal house is subject to agreement number B75-15-05 from the prefecture of Police. This includes provision for supervision by the internal ethics committee for animal experimentation of the Pasteur Institute.

### Gene Targeting

Briefly, a region encompassing exons 2, 3 and 4 has been flanked by *loxP* sites. Under Cre recombinase activity, this sequence should be deleted, and since it contains the transmembrane as well as most of the Brichos domain, the transcript generated from the recombined allele should lead to a truncated non-functional protein. The reporter cassette has been inserted in the 3′ flanking region of exon 6 and an *IRES* (Internal-Ribosomal-Entry-Site) sequence has been used to allow the formation of a bi-cistronic RNA. The *Neo* selection cassette is flanked by FRT sites, allowing for its deletion by Flip recombinase activity.

The *Itm2a* gene is located in the syngenic region of the X chromosome [46]. We decided therefore to apply the following code for naming the different genotypes: *Itm2a^XcdKON/Y^* for males (no Cre, no Flip) and *Itm2a^XcdKON/X^* for females, sometimes simplified as *Itm2a^cdKON/+^*. After Neo removal, the conditional allele is named *cd* (cd, for conditional), and after Cre recombinase deletion, this leads to a knock-out allele, named KO.

A total number of 949 embyonic stem (ES) cell clones were tested for homologous recombination, using a 5′ probe, by the Southern blotting technique as a first test (data not shown). Positive 5′-tested clones were then checked using a 3′ probe, but the results were unsatisfatory. Therefore homologous recombination in the 3′ region was checked by long template PCR. Two clones were correctly recombined, and one of them was injected into blastocysts obtained from C57/Bl6:DBA2 F1 mice. Chimera were obtained and their progeny (F0) were then tested in the 3′ recombined region using long template DNA, to demonstrate that the recombined allele has been correctly transmitted to the germ line. Heterozygote mice were then intercrossed with C57/Bl6 mice for 4–8 generations.

### Breeding Mutant Mice and Genotyping

The following mouse lines were used: *Pax3^PAX3-FKHR-IRESnlaZ/+^* (referred to as *Pax3^PAX3-FKHR/+^*), *Pax3^Pax3-En-IRESnLacZ/+^* (referred to as *Pax3^Pax3-En/+^*), *Myf5^nLacZ/+^*, *Pax3^nLacZ/+^*, *Pax3^GFP/+^* and *Pax3^Cre/+^*. Embryos were genotyped as described previously: *Pax3^PAX3-FKHR^* and *Pax3^nLacZ^* [21], *Pax3^Pax3-En^* [11], *Pax3^GFP/+^* [2] and *Pax3^Cre/+^* [40]. *Pax3* lines have been maintained in a C57/Bl6 background. *Myf5* mutant mice are maintained in a mixed background.

### Dissection and Embryo Preparation

Embryos were collected after natural overnight mating and dated, taking Embryonic day (E) 0.5 as the day after the appearance of the vaginal plug. Briefly, embryos were fixed in 4% para-formaldehyde at 4°C, overnight for *in situ* hybridization, 2 hours for immuno-detection and 15 minutes for X-Gal staining.

### In situ Hybridization

Whole-mount *in situ* hybridizations with digoxigenin-labeled probes were performed as described in [31]. The *Itm2a* probe was synthesized using the image clone 3469636 (Open Biosystems) and linearized by BamH1 enzyme digestion.

### Real-time PCR

All PCR reactions were carried out in duplicate (triplicate for the standard curves) using the iQ™ SYBR® Green Supermix (Bio-Rad) [15] and an iCycler (Bio-Rad) thermal cycler.

### Chromatin Immunoprecipitation

Pax3 ChIP was performed on trunk and limb cells that had been crosslinked with formaldehyde from the *P34* transgenic line at E11.5, as described in [15].

The previously identified binding site for Pax3, located within the first intron of *Itm2a* [19] was tested for *in vivo* Pax3 binding using ChIP-qPCR with the primers listed below.

*Itm2a*: forward: TTTGTGAGATTCGGTGTAGTTGA

Reverse: TTCAGAGAAGCGGCAATAGAA

Primers for *P34* and *Myf5* sequences are described in [15].

### Immunohistochemistry

Fluorescent co-immunohistochemistry on sections was carried out as described previously [15]. The following antibodies were used: anti-Pax3 (monoclonal, DSHB, dilution 1/250), anti-MF20 (monoclonal, DSHB, 1/250), anti-laminin (polyclonal, Sigma, 1/200), anti-slow myosin (monoclonal, Sigma, 1/1000), anti Sox9 (R&D Systems, 1∶100) and anti-ß-Galactosidase (polyclonal, provided by Dr. J.-F Nicolas (France), 1/500). Secondary antibodies were coupled to fluorochromes: Alexa 488 (Molecular probes, 1/500) and Alexa 546 (Molecular probes, 1/1500). Images were obtained with an Apotome Zeiss microscope and Axiovision software at the Pasteur imaging center (PFID). All images were assembled in Adobe Photoshop.

### Single Fiber Preparation and Regeneration Assays

Single fibers were prepared, cultured and processed for immunostaining as described previously [44]. After dissection, muscles were directly frozen in isopentane. Cryosections (10 microns) were processed for ß-Gal staining and immunostaining as described in [44]. Muscle injury using cardiotoxin injection and regeneration assays were performed, as described in [44].

## Supporting Information

Figure S1 **X-Gal staining of a** ***Pax3^Pax3-En/+^*** **embryo at E10.75.** A close-up view of the interlimb somitic region is shown in the right panel. X-Gal staining indicates that myogenic progenitor cells are still present in the presence of Pax3-Engrailed. (*Pax3-En* stands for *Pax3-Engrailed-Ires-nlacZ*).
(TIF)

Figure S2 **Sections of the whole-mount** ***in situ*** **hybridization (ISH) shown in** **Figure 1** **, panels G, H.** The level of the section is indicated by a white bar and labeled A, B, C for interlimb, hindlimb and caudal level somites respectively. Left hand panels correspond to the wild-type embryo shown in G and right hand panels to the mutant shown in H. *Itm2a* is over-expressed in the presence of PAX3-FKHR.
(TIF)

Figure S3 **A–B, Whole mount** ***in situ*** **hybridization (ISH) with an** ***Itm2a*** **antisense riboprobe on control** ***Pax3^Pax7-IRESnLacZ/+^*** **(** ***Pax3^Pax7/+^*** **) (A) and mutant** ***Pax3^Pax7-IRESnLacZ/Splotch^*** **(** ***Pax3^Pax7/Sp^*** **) (B) embryos at E11.5.** *Pax3^Sp^* is a naturally occuring mutant allele. C–D, X-Gal staining of control *Pax3^Pax7-IRESnLacZ/+^* (C) and mutant *Pax3^Pax7-IRESnLacZ/Sp^* (B) embryos at E11.5. Close-ups of the interlimb somite region are shown. X-Gal staining indicates that myogenic progenitor cells (normally, Pax3^+^) are still present, although reduced in the limb buds where Pax3 plays a critical role in cell migration from the somite. Red arrows point to myogenic sites of *Itm2a* expression.
(TIF)

Figure S4 **A–D’, Whole mount** ***in situ*** **hybridization (ISH) with an** ***Itm2a*** **antisense riboprobe on** ***Myf5^nLacZ/+^*** **(A, C, A’, C’) and** ***Myf5^nLacZ/nLacZ^*** **(B, D, B’, D’) embryos.** A’–D’ show close-ups of the interlimb somite region. Embryonic stages are as indicated. Blue arrows point to *Itm2a* expression in the somites.
(TIF)

Figure S5 **X-Gal staining of an E10.5** ***Itm2a^XKO/Y^*** **male (A),** ***Itm2a^XKO/XKO^*** **(B) and** ***Itm2a^XKO/X^*** **(C) female embryos at E10.5.** Note the chimeric X-Gal staining in the female embryo, due to random X inactivation in somatic cells.
(TIF)

Figure S6 **Immunohistochemistry on transverse sections through forelimb buds of an** ***Itm2a^XcdKO/X^; Pax3^Cre/+^*** **embryo at E10.5, using antibodies to ßGal (green) and the chondrocyte marker Sox9 (red).** A, B,C: 20X, C is the merged image showing co-expression. A’, B, C’ : 63X of the region highlighted in A,B and C.
(TIF)

Figure S7 **Whole mount in situ hybridization (ISH) for** ***Itm2a*** **transcripts in wild-type (A), heterozygote** ***Itm2a^XKO/X^*** **(B) and mutant** ***Itm2a^XKO/Y^*** **(C) embryos at E11.5.** In B and C the mice were crossed with the *PGK-Cre* line to delete the floxed *Itma2* allele. The signal in the head is propably a mixture of in situ hybridization background signal and some *Itm2a* expression in the most anterior region.
(TIF)

Figure S8 **X-Gal staining of control** ***Pax3^GFP/GFP^*** **;** ***Itm2a^XcdKO/Y^*** **(A) and** ***Pax3^GFP/GFP^*** **;** ***Itm2a^KO/Y^*** **mutant (B) embryos at E10.5.** The mice were crossed with the *PGK-Cre* line to delete the floxed *Itma2* allele.
(TIF)
