# Supplementary figures and images for: Itm2a Is a Pax3 Target Gene, Expressed at Sites of Skeletal Muscle Formation In Vivo
Source: PLoS One. 2013 May 1;8(5):e63143. doi: 10.1371/journal.pone.0063143 (PMC3641095; doi:10.1371/journal.pone.0063143)

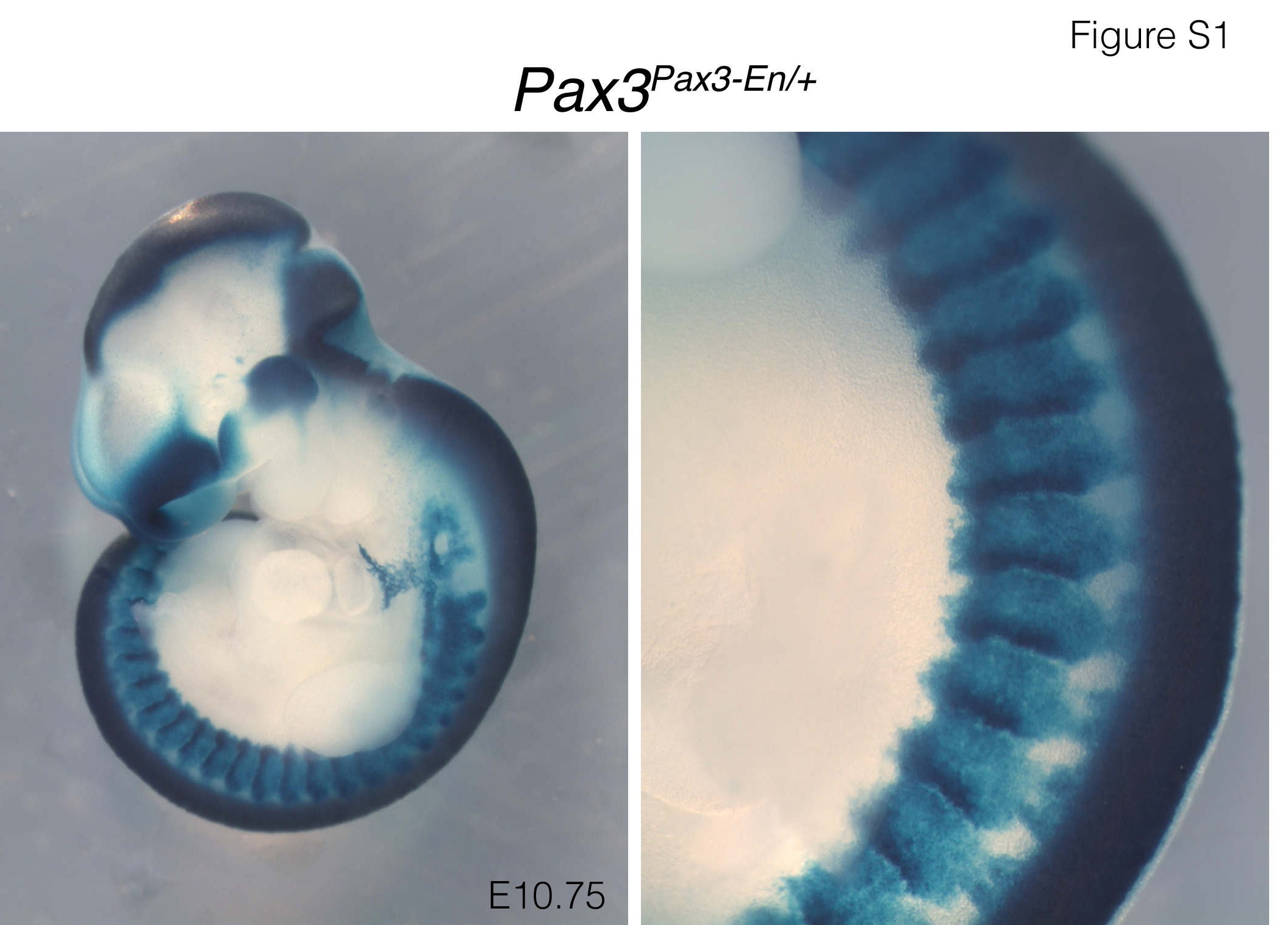

Supplement: Figure S1 — X-Gal staining of a Pax3Pax3-En/+ embryo at E10.75. A close-up view of the interlimb somitic region is shown in the right panel. X-Gal staining indicates that myogenic progenitor cells are still present in the presence of Pax3-Engrailed. (Pax3-En stands for Pax3-Engrailed-Ires-nlacZ). (TIF) [file pone.0063143.s001.tif]

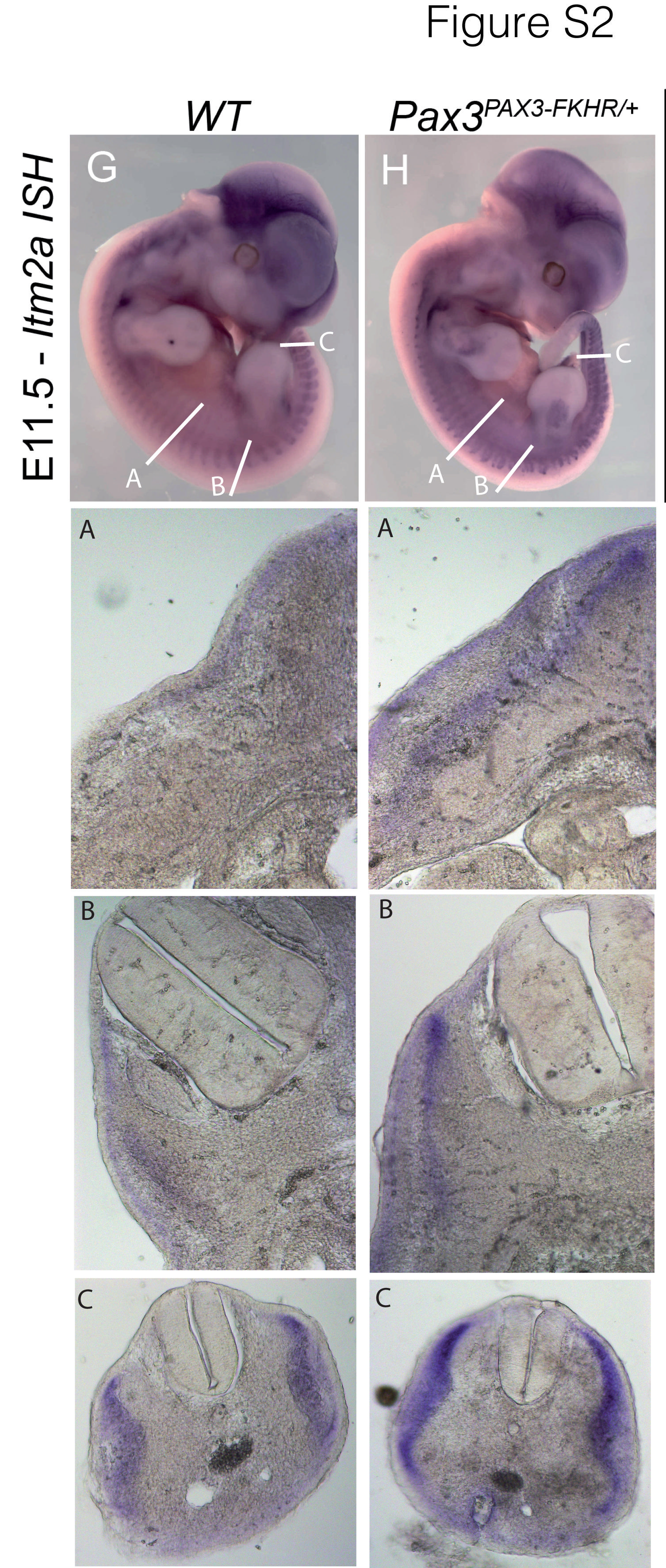

Supplement: Figure S2 — Sections of the whole-mount in situ hybridization (ISH) shown in Figure 1 , panels G, H. The level of the section is indicated by a white bar and labeled A, B, C for interlimb, hindlimb and caudal level somites respectively. Left hand panels correspond to the wild-type embryo shown in G and right hand panels to the mutant shown in H. Itm2a is over-expressed in the presence of PAX3-FKHR. (TIF) [file pone.0063143.s002.tif]

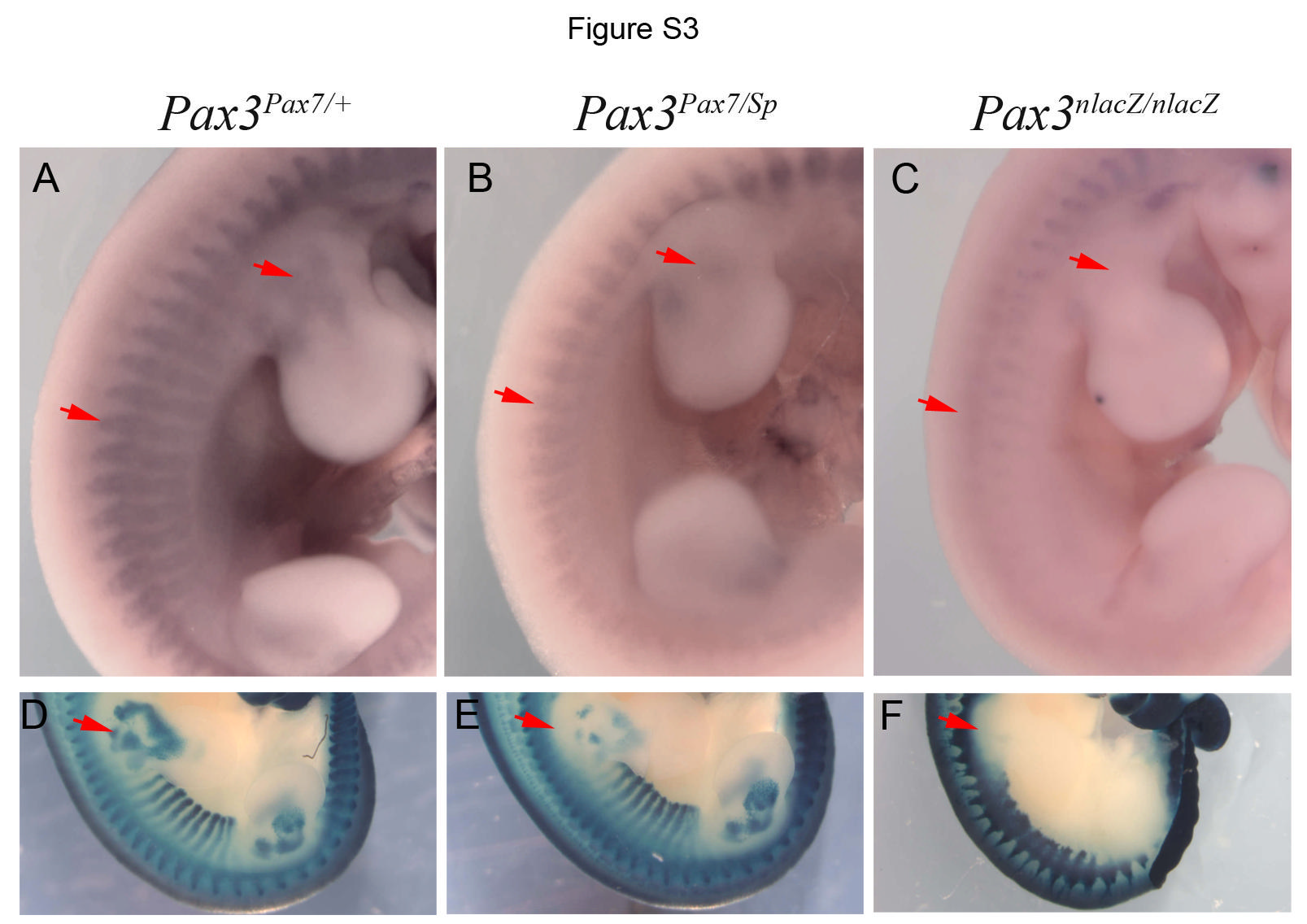

Supplement: Figure S3 — A–B, Whole mount in situ hybridization (ISH) with an Itm2a antisense riboprobe on control Pax3Pax7-IRESnLacZ/+ ( Pax3Pax7/+ ) (A) and mutant Pax3Pax7-IRESnLacZ/Splotch ( Pax3Pax7/Sp ) (B) embryos at E11.5. Pax3Sp is a naturally occuring mutant allele. C–D, X-Gal staining of control Pax3Pax7-IRESnLacZ/+ (C) and mutant Pax3Pax7-IRESnLacZ/Sp (B) embryos at E11.5. Close-ups of the interlimb somite region are shown. X-Gal staining indicates that myogenic progenitor cells (normally, Pax3+) are still present, although reduced in the limb buds where Pax3 plays a critical role in cell migration from the somite. Red arrows point to myogenic sites of Itm2a expression. (TIF) [file pone.0063143.s003.tif]

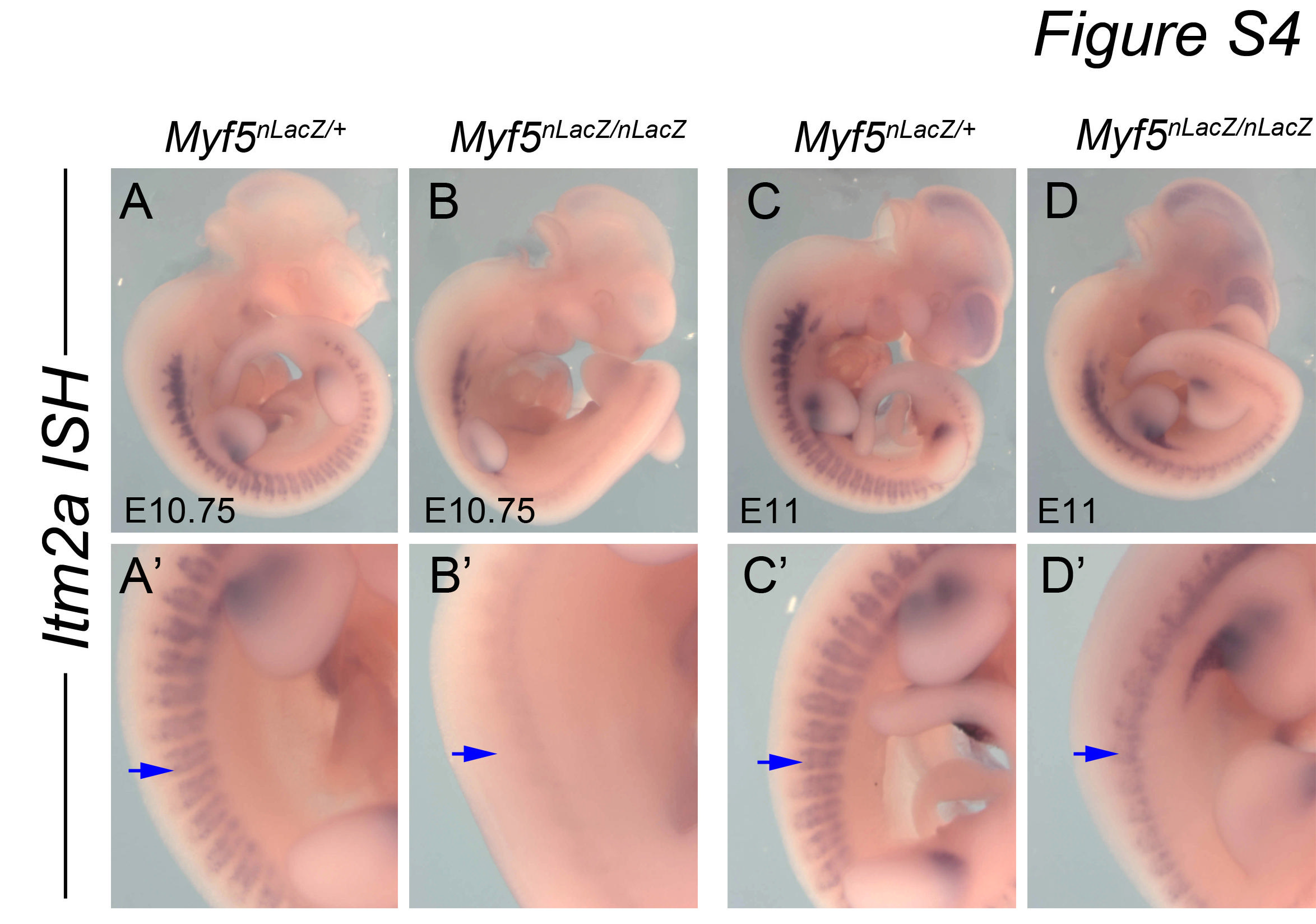

Supplement: Figure S4 — A–D’, Whole mount in situ hybridization (ISH) with an Itm2a antisense riboprobe on Myf5nLacZ/+ (A, C, A’, C’) and Myf5nLacZ/nLacZ (B, D, B’, D’) embryos. A’–D’ show close-ups of the interlimb somite region. Embryonic stages are as indicated. Blue arrows point to Itm2a expression in the somites. (TIF) [file pone.0063143.s004.tif]

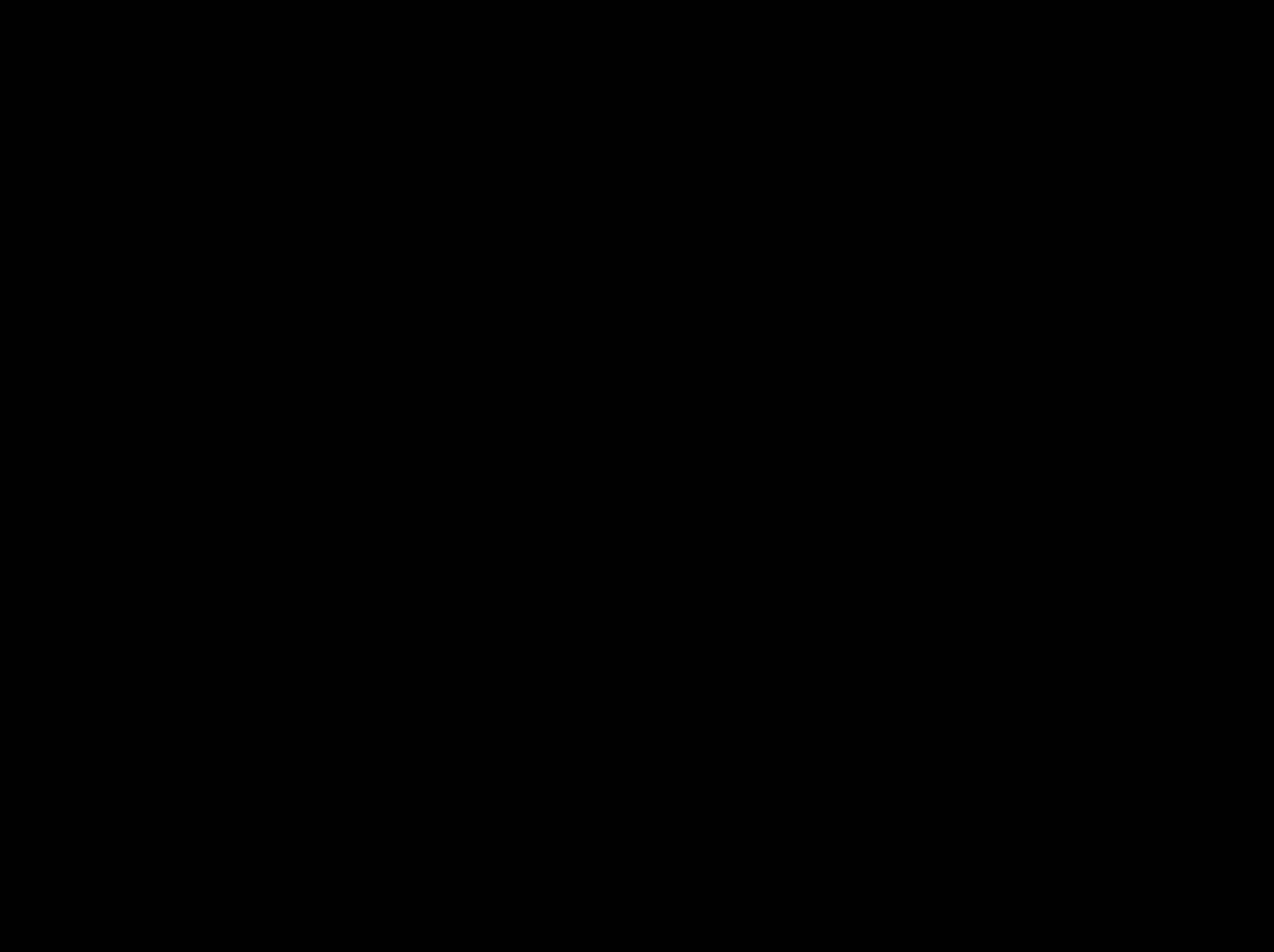

Supplement: Figure S5 — X-Gal staining of an E10.5 Itm2aXKO/Y male (A), Itm2aXKO/XKO (B) and Itm2aXKO/X (C) female embryos at E10.5. Note the chimeric X-Gal staining in the female embryo, due to random X inactivation in somatic cells. (TIF) [file pone.0063143.s005.tif]

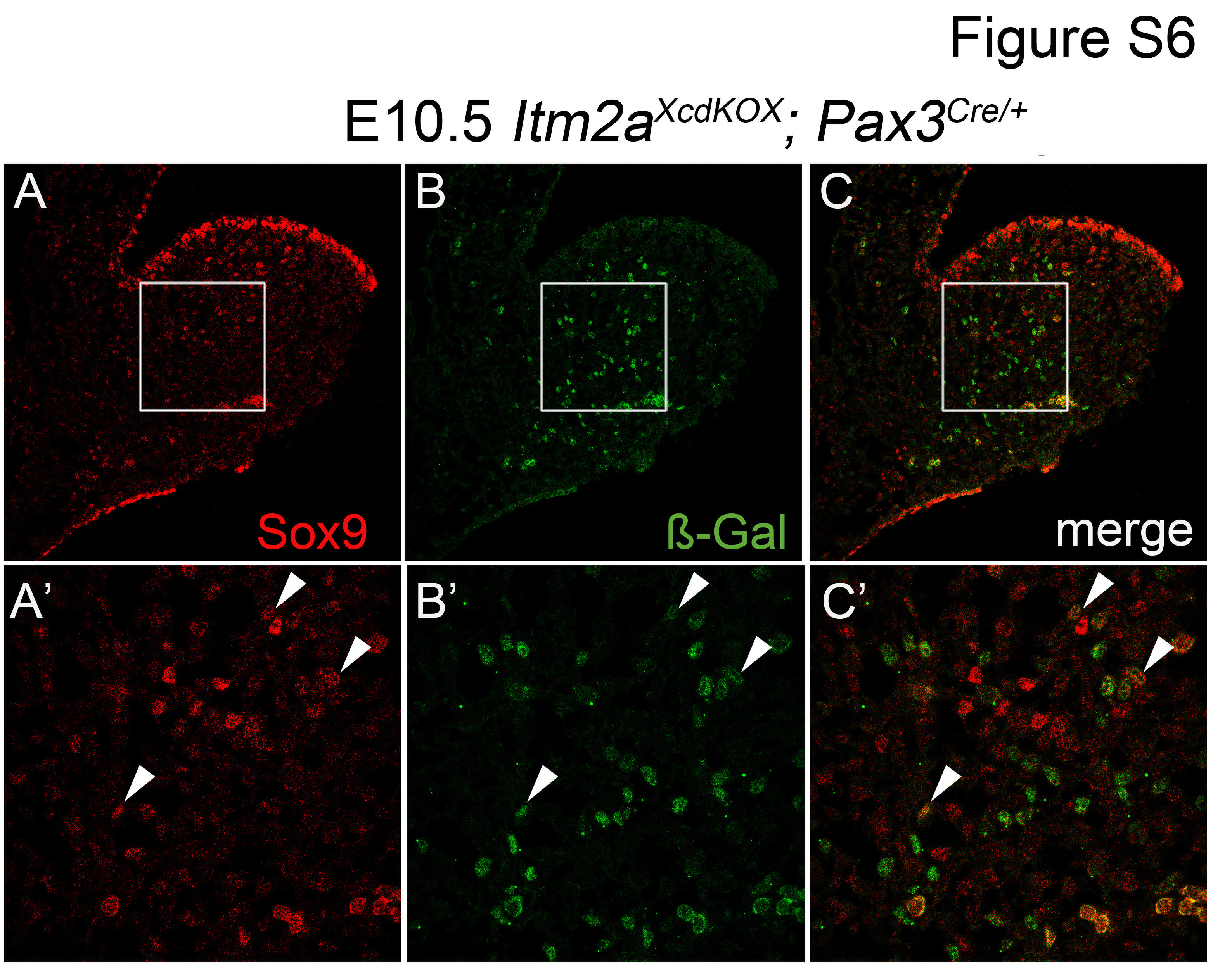

Supplement: Figure S6 — Immunohistochemistry on transverse sections through forelimb buds of an Itm2aXcdKO/X; Pax3Cre/+ embryo at E10.5, using antibodies to ßGal (green) and the chondrocyte marker Sox9 (red). A, B,C: 20X, C is the merged image showing co-expression. A’, B, C’ : 63X of the region highlighted in A,B and C. (TIF) [file pone.0063143.s006.tif]

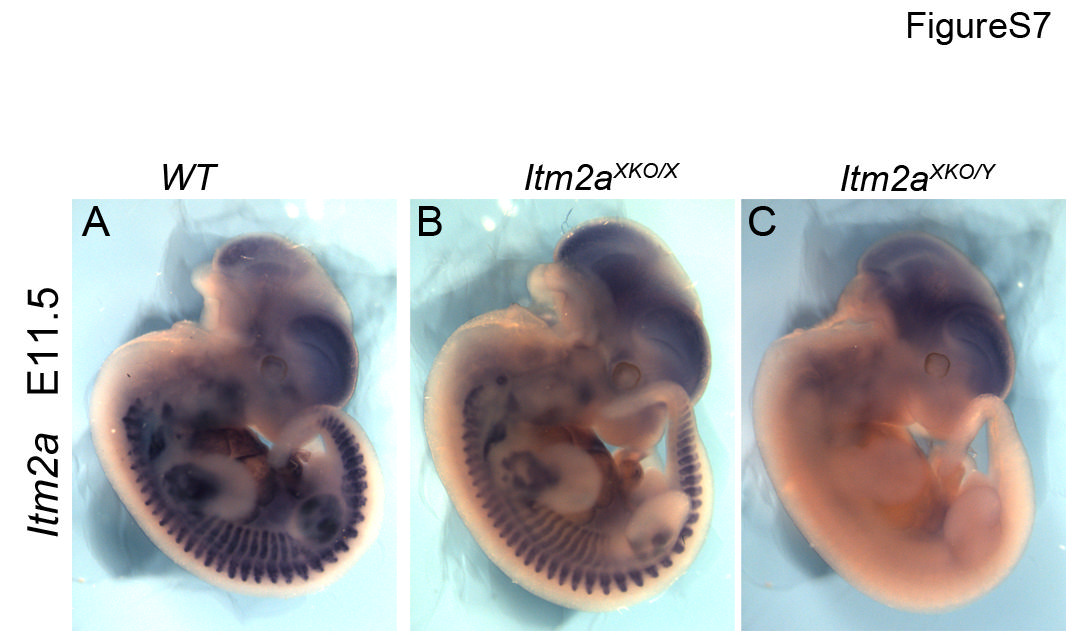

Supplement: Figure S7 — Whole mount in situ hybridization (ISH) for Itm2a transcripts in wild-type (A), heterozygote Itm2aXKO/X (B) and mutant Itm2aXKO/Y (C) embryos at E11.5. In B and C the mice were crossed with the PGK-Cre line to delete the floxed Itma2 allele. The signal in the head is propably a mixture of in situ hybridization background signal and some Itm2a expression in the most anterior region. (TIF) [file pone.0063143.s007.tif]

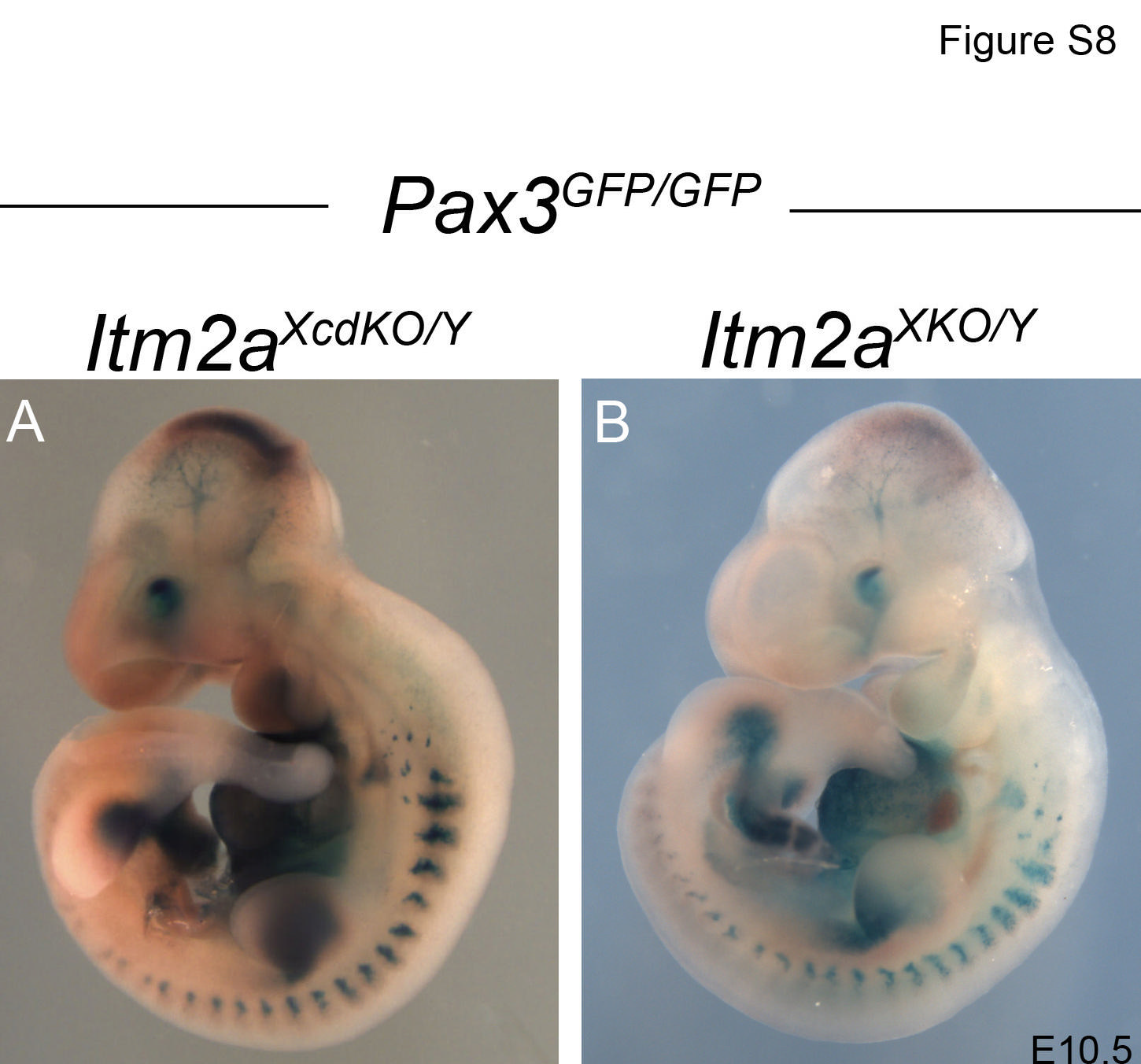

Supplement: Figure S8 — X-Gal staining of control Pax3GFP/GFP ; Itm2aXcdKO/Y (A) and Pax3GFP/GFP ; Itm2aKO/Y mutant (B) embryos at E10.5. The mice were crossed with the PGK-Cre line to delete the floxed Itma2 allele. (TIF) [file pone.0063143.s008.tif]
